# Supplementary material for: The lung microbiota in nontuberculous mycobacterial pulmonary disease
Source: PLoS One. 2023 May 26;18(5):e0285143. doi: 10.1371/journal.pone.0285143 (PMC10218745; doi:10.1371/journal.pone.0285143)
Supplement: S1 Fig — (DOCX) [file pone.0285143.s001.docx]

**[Supporting information]**

**The lung microbiota in nontuberculous mycobacterial pulmonary disease**

Bo-Guen Kim^1^**^¶^**, Noeul Kang^1^**^¶^**, Su-Young Kim^1^, Dae Hun Kim^1*^, Hojoong Kim^1^, O Jung Kwon^1^, Hee Jae Huh^2^, Nam Yong Lee^2^, Byung Woo Jhun^1*^

^1^Division of Pulmonary and Critical Care Medicine, Department of Medicine, Samsung Medical Center, Sungkyunkwan University School of Medicine, Seoul, South Korea;

^2^Department of Laboratory Medicine and Genetics, Samsung Medical Center, Sungkyunkwan University School of Medicine, Seoul, South Korea

**S1 Fig.** Study samples.

**
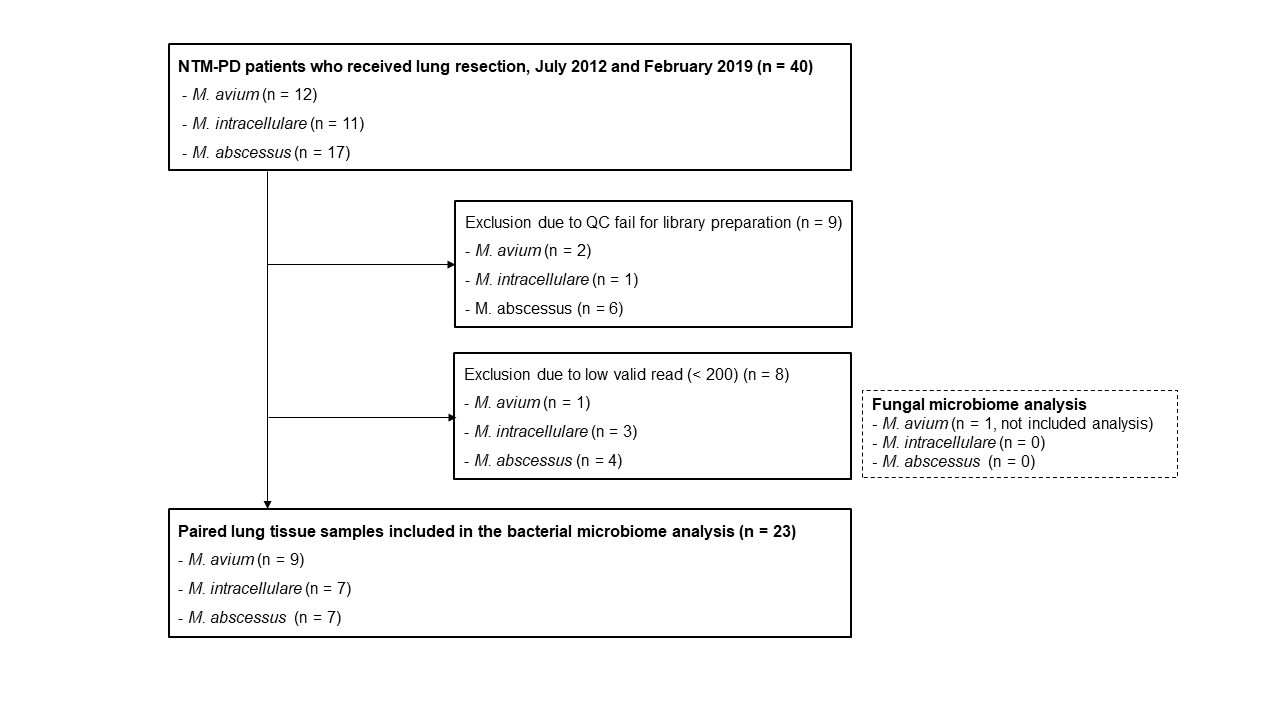
**
