# Supplementary material for: The lung microbiota in nontuberculous mycobacterial pulmonary disease
Source: PLoS One. 2023 May 26;18(5):e0285143. doi: 10.1371/journal.pone.0285143 (PMC10218745; doi:10.1371/journal.pone.0285143)

**S2 Fig.** Taxonomic relative abundance of phyla compared between involved and non-involved sites. Taxa <1% are not indicated.

**2-1.** *Proteobacteria* (Wilcoxon rank-sum test, p=0.606)


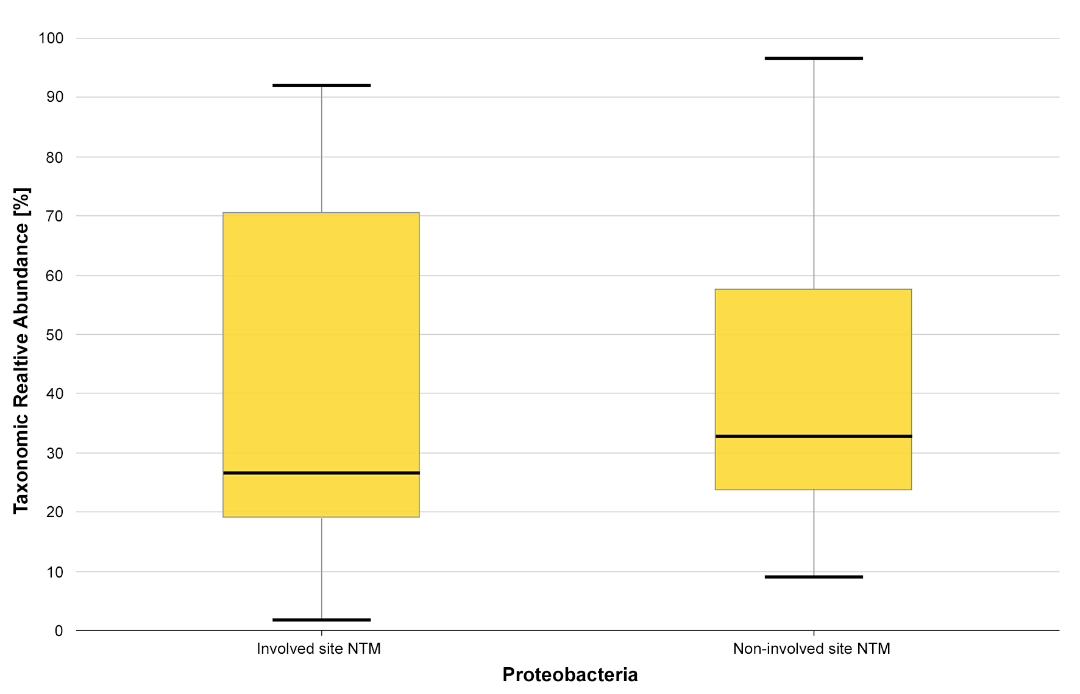


**2-2***. Acidobacteria* (Wilcoxon rank-sum test, p=0.085)
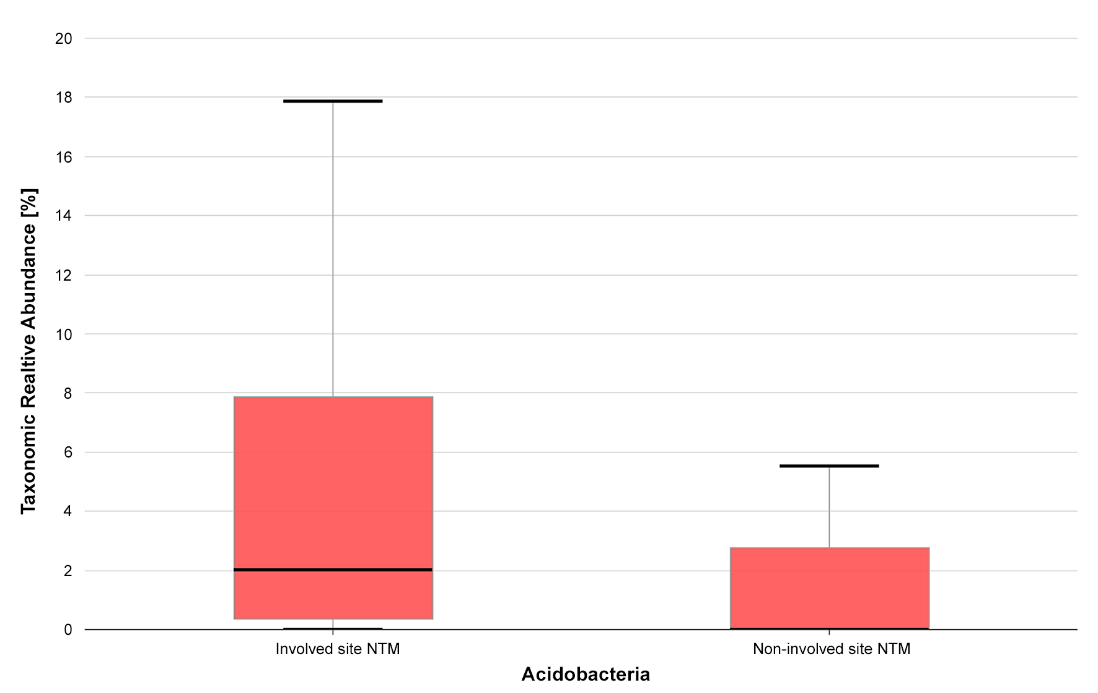


**2-3.** *Chloroflexi* (Wilcoxon rank-sum test, p=0.253)
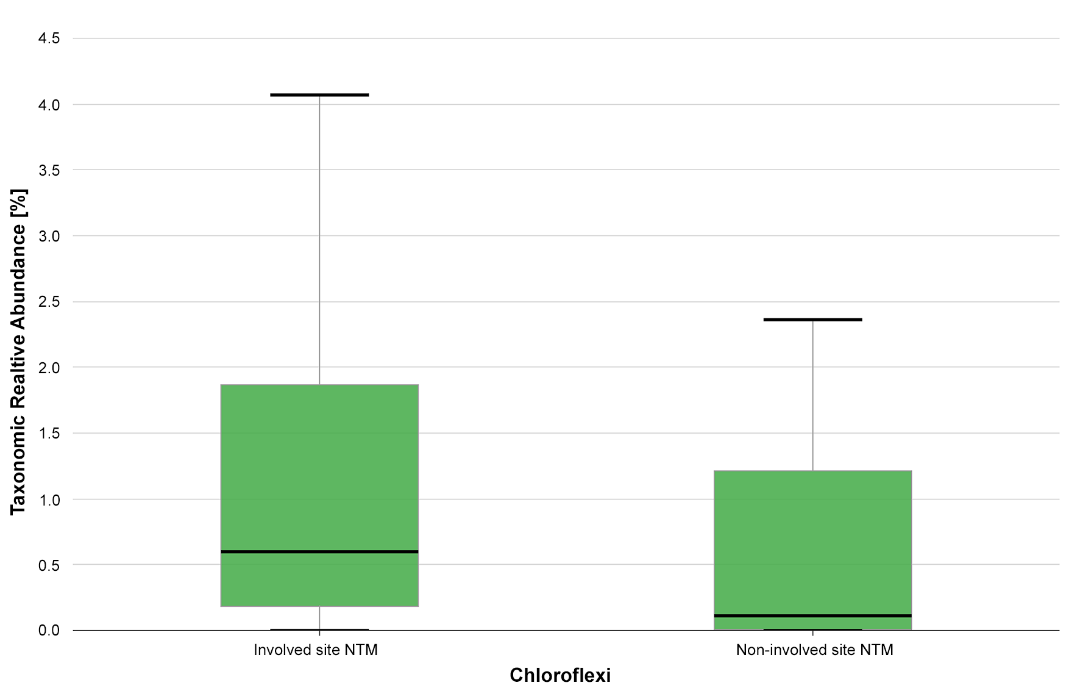


**2-4.** *Actinobacteria* (Wilcoxon rank-sum test, p=0.307)
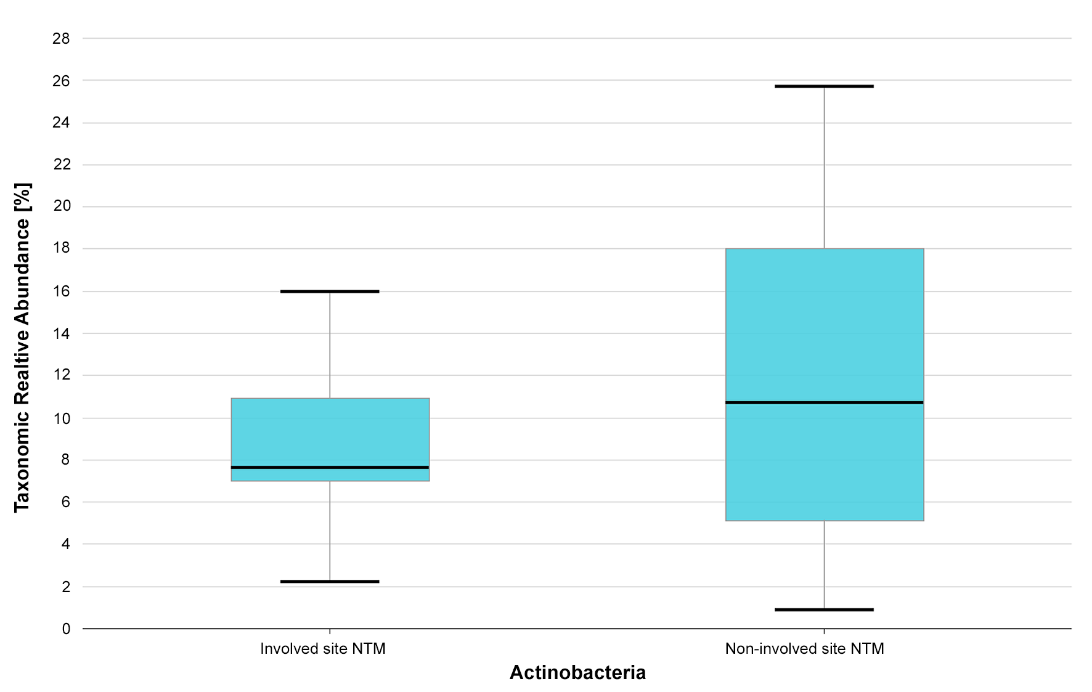


**2-5.** *Bacteroidetes* (Wilcoxon rank-sum test, p=0.668)


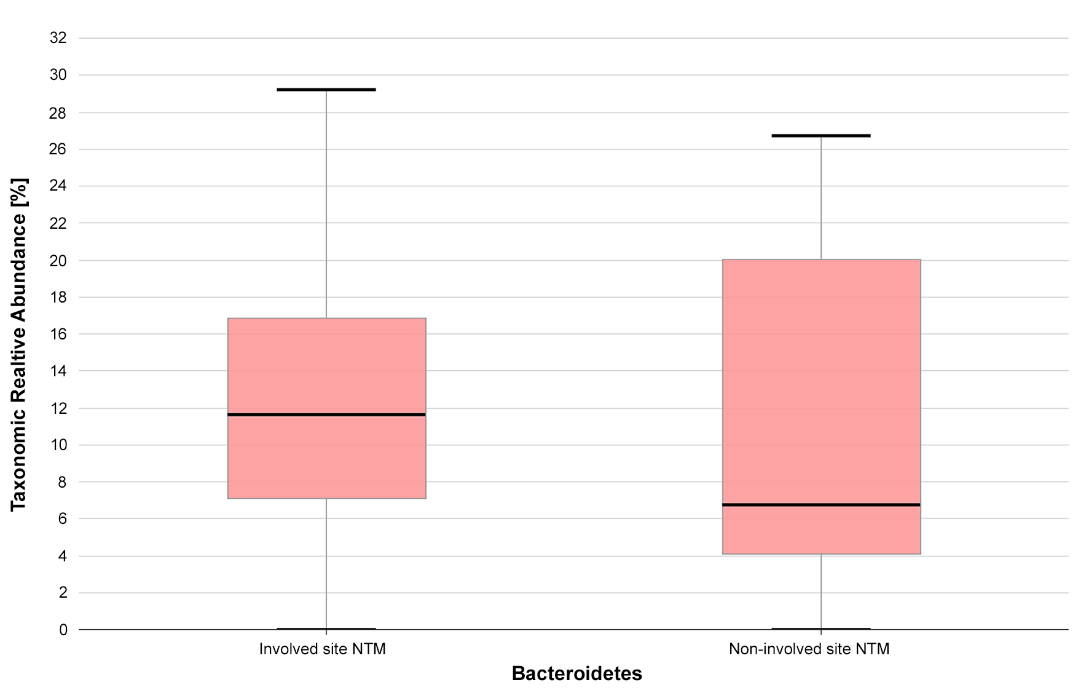


**2-6.** *Firmicutes* (Wilcoxon rank-sum test, p=0.386)


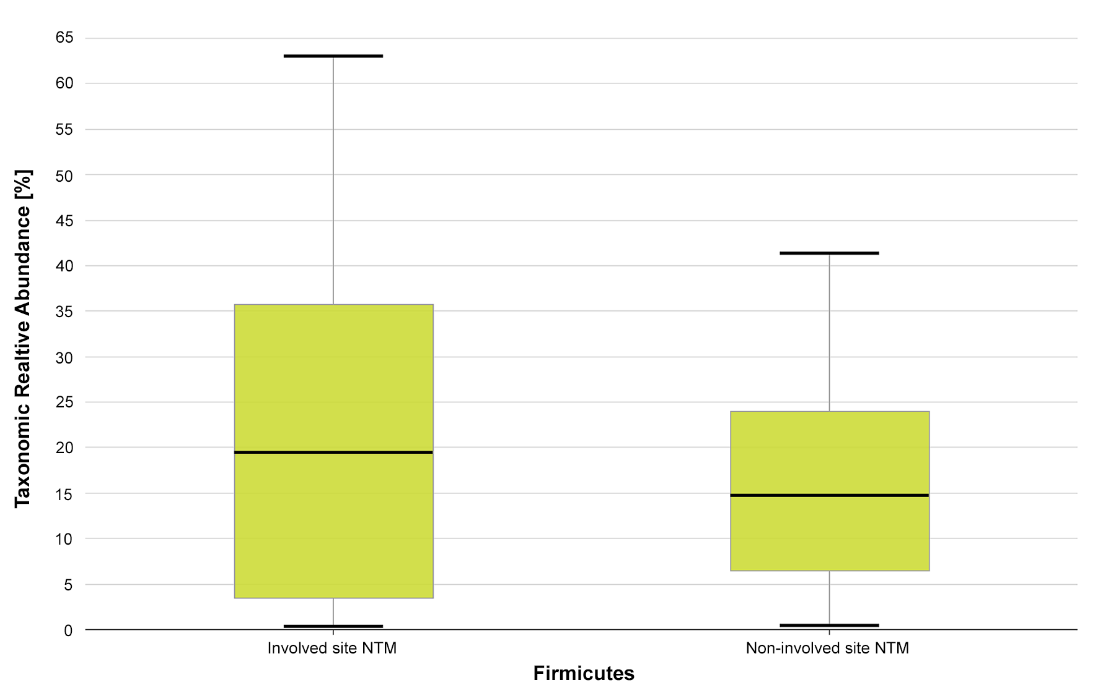


**2-7.** *Verrucomicrobia* (Wilcoxon rank-sum test, p=0.010)


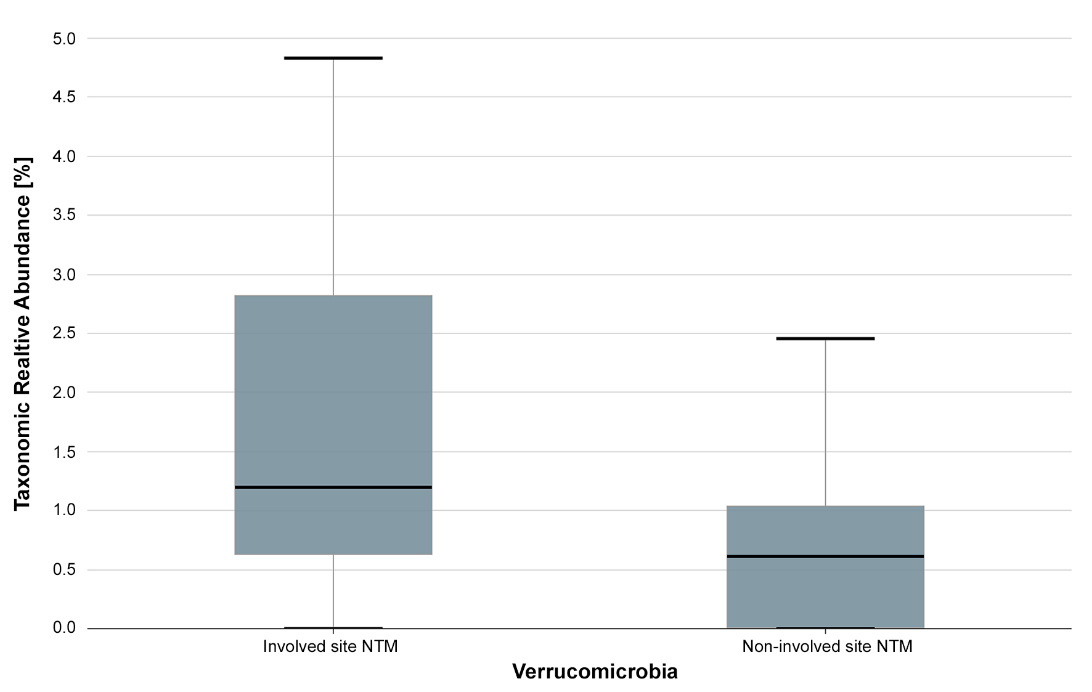

Supplement: S2 Fig — Taxa <1% are not indicated. (DOCX) [file pone.0285143.s002.docx]
