# Supplementary material for: The lung microbiota in nontuberculous mycobacterial pulmonary disease
Source: PLoS One. 2023 May 26;18(5):e0285143. doi: 10.1371/journal.pone.0285143 (PMC10218745; doi:10.1371/journal.pone.0285143)

**S3 Fig.** Taxonomic relative abundance of genera compared between involved and non-involved sites. Taxa <1% are not indicated.

**3-1.** *Rahnella* (Wilcoxon rank-sum test, p=0.002)


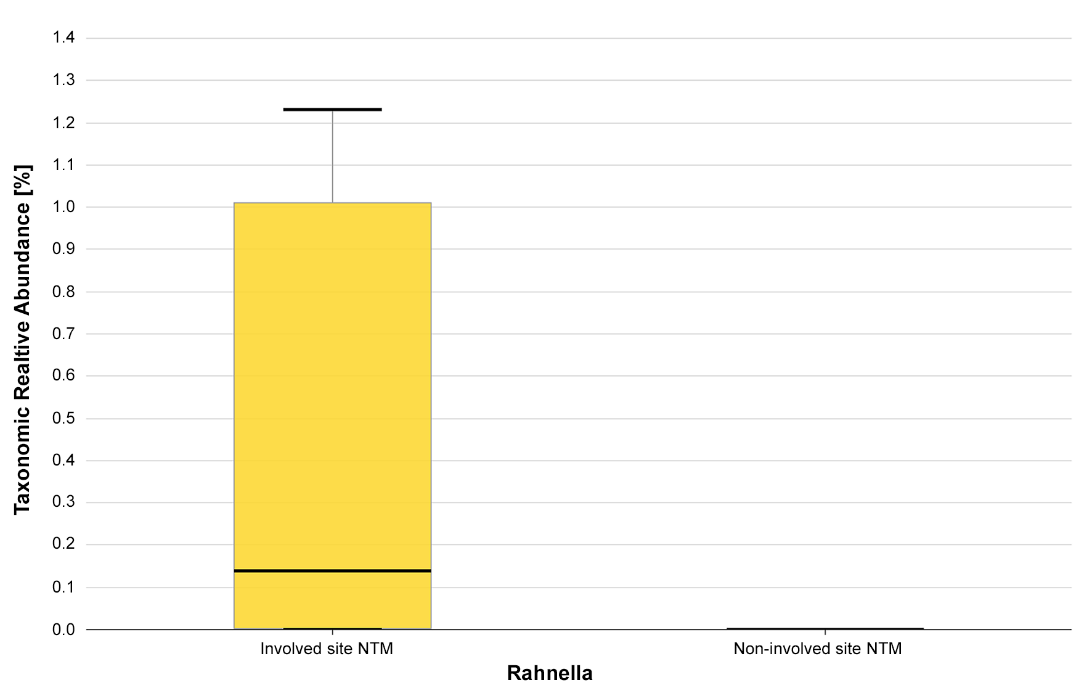


**3-2.** *Aquabacterium* (Wilcoxon rank-sum test, p=0.046)
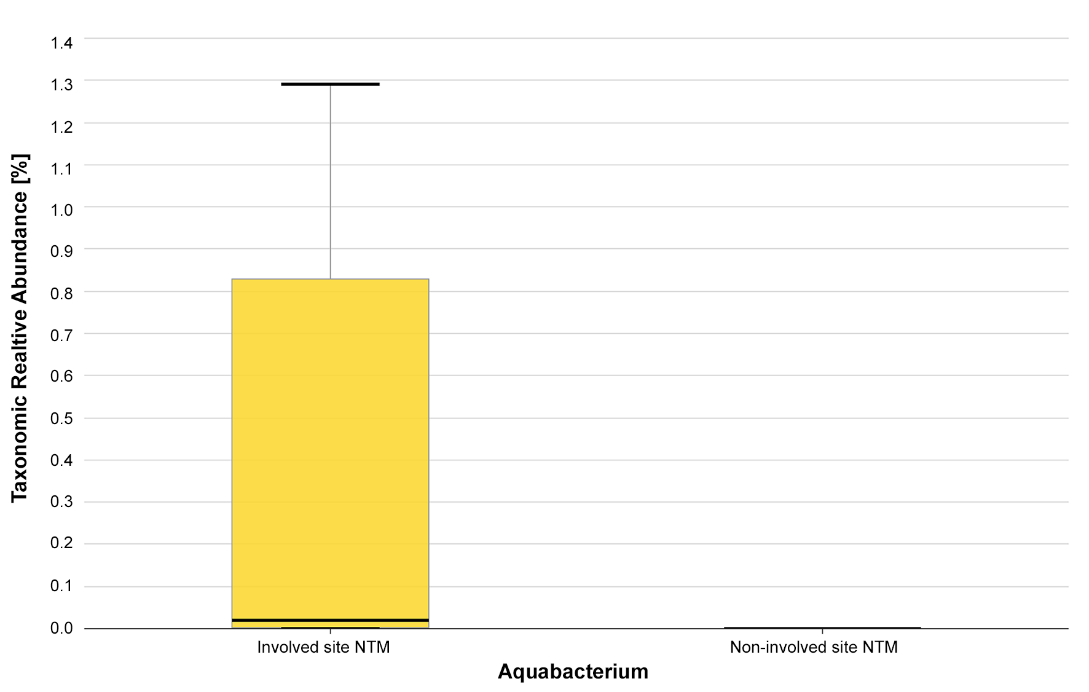


**3-3. *Oscillibacter*** (Wilcoxon rank-sum test, p=0.005)


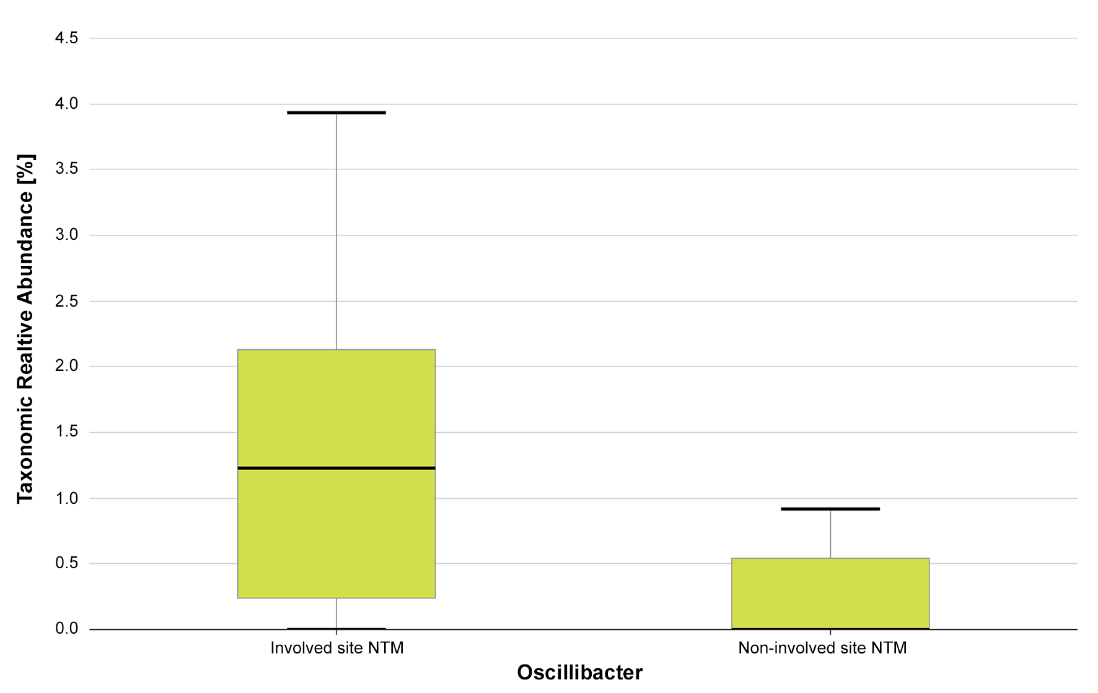


**3-4. *Limnohabitans*** (Wilcoxon rank-sum test, p=0.003)
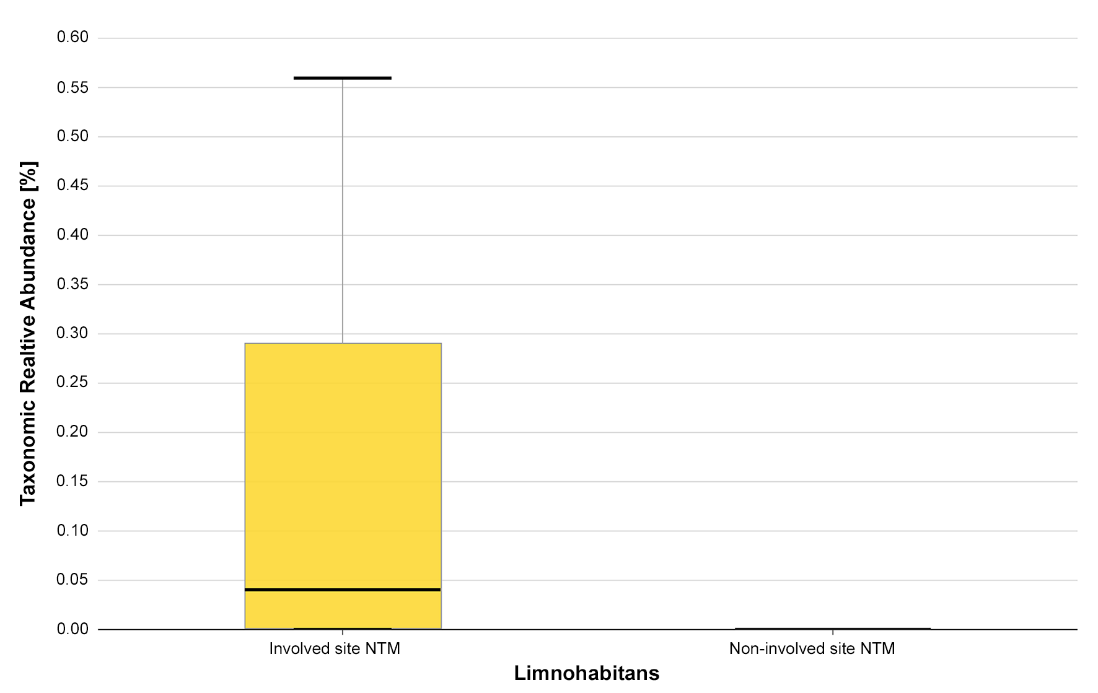


**3-5. *Ruminococcus*** (Wilcoxon rank-sum test, p=0.006)


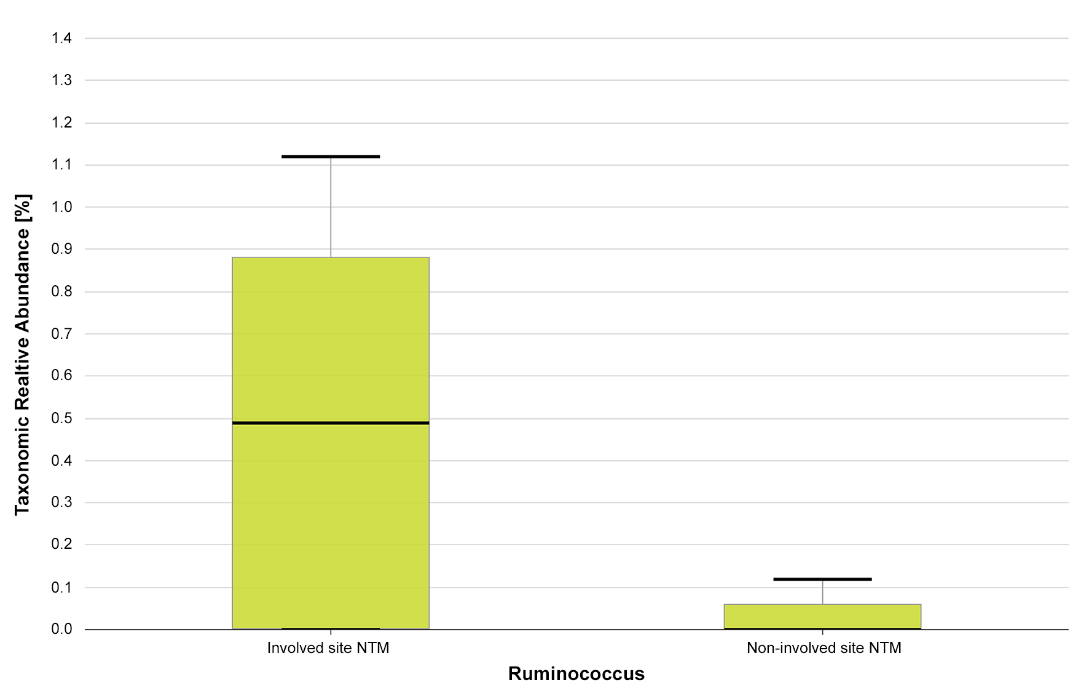


**3-6. *Blautia*** (Wilcoxon rank-sum test, p=0.026)
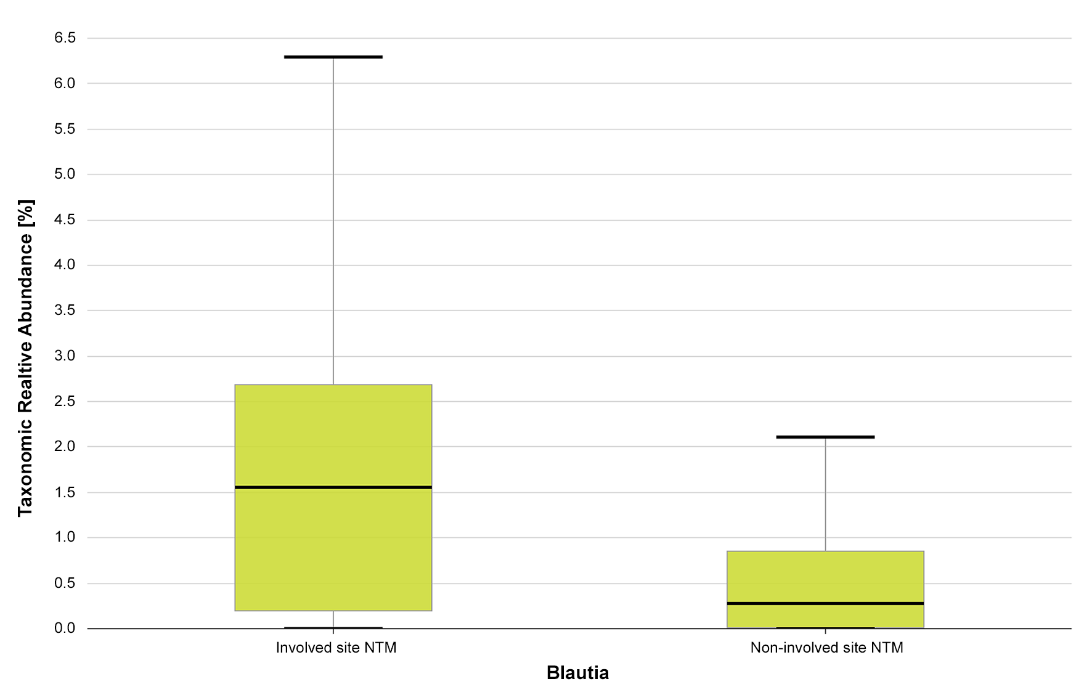


**3-7. *Faecalibacterium*** (Wilcoxon rank-sum test, p=0.011)


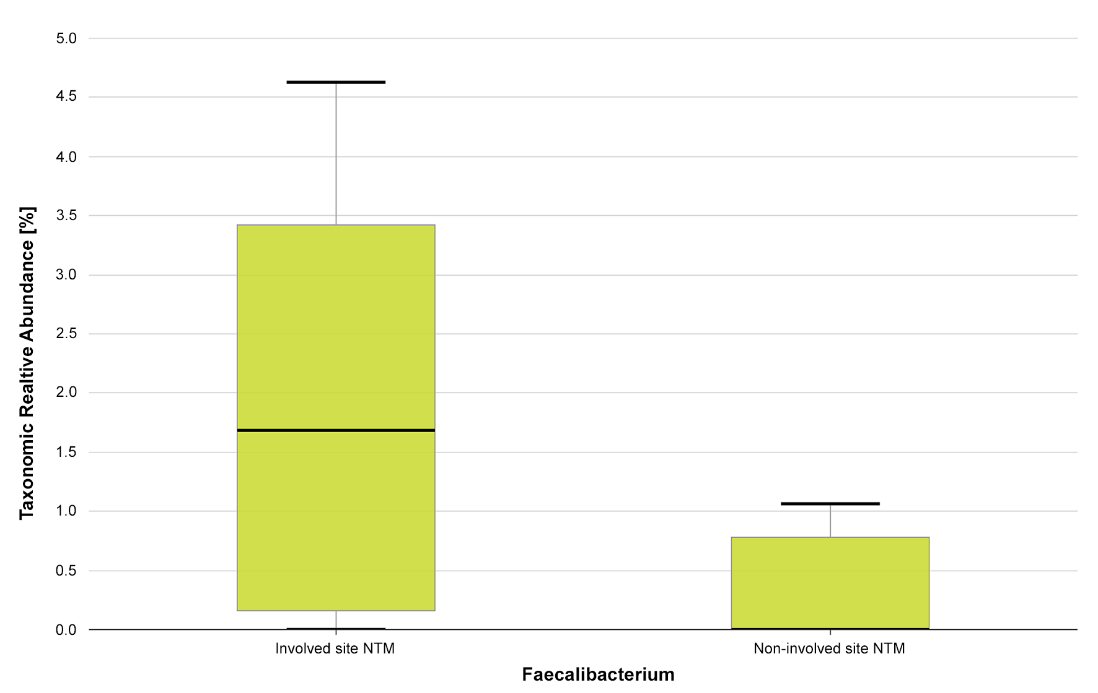


**3-8. *Acinetobacter*** (Wilcoxon rank-sum test, p=6.5e-7)
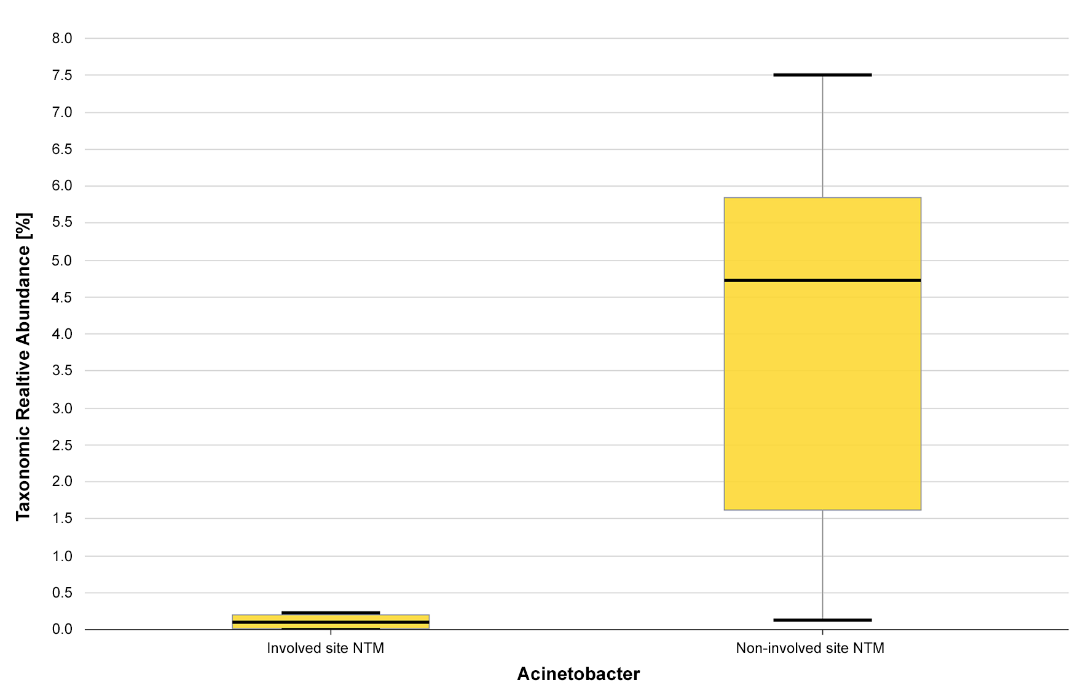


**3-9. *Enhydrobacter*** (Wilcoxon rank-sum test, p= 0.022)


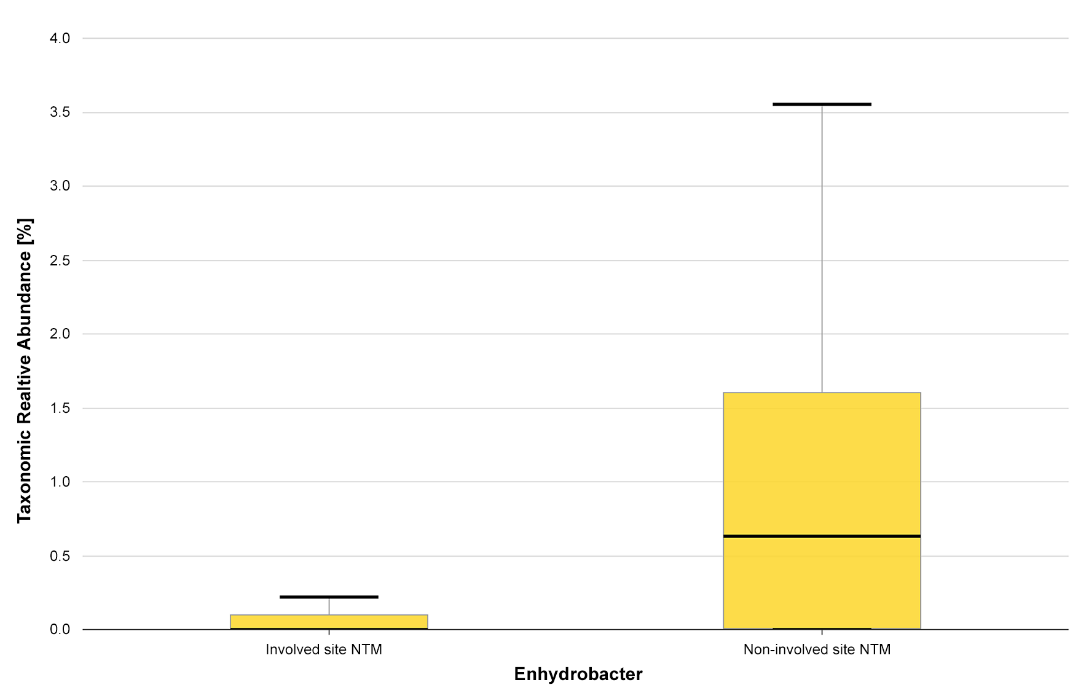

Supplement: S3 Fig — Taxa <1% are not indicated. (DOCX) [file pone.0285143.s003.docx]
