# Supplementary material for: The lung microbiota in nontuberculous mycobacterial pulmonary disease
Source: PLoS One. 2023 May 26;18(5):e0285143. doi: 10.1371/journal.pone.0285143 (PMC10218745; doi:10.1371/journal.pone.0285143)
Supplement: S1 Table — (DOCX) [file pone.0285143.s004.docx]

**S1 Table.** Library data on lung tissue samples included in the study.

| **Sample Name** | **Valid reads** | **OTUs** | **Good's coverage of library (%)** |
| --- | --- | --- | --- |
| **Involved sites** | | | |
| Mavi-1(FC)-B | 29401 | 879 | 99.41 |
| Mavi-2(NB)-B | 1321 | 258 | 96.52 |
| Mavi-3(FC)-B | 4450 | 637 | 97.19 |
| Mavi-4(NB)-B | 7407 | 1126 | 96.75 |
| Mavi-5(FC)-B | 8632 | 565 | 98.59 |
| Mavi-7(FC)-B | 2799 | 245 | 97.96 |
| Mavi-8(FC)-B | 1440 | 151 | 96.74 |
| Mavi-9(NB)-B | 221 | 33 | 97.29 |
| Mavi-10(NB)-B | 305 | 23 | 99.02 |
| Mint-2(NB)-B | 7470 | 959 | 97.54 |
| Mint-4(FC)-B | 10823 | 725 | 98.71 |
| Mint-5(FC)-B | 4224 | 381 | 99.36 |
| Mint-6(FC)-B | 4284 | 555 | 97.41 |
| Mint-7(FC)-B | 9444 | 1025 | 97.35 |
| Mint-8(NB)-B | 7455 | 954 | 97.48 |
| Mint-10(FC)-B | 1045 | 16 | 99.62 |
| Mabs-3(FC)-B | 4271 | 442 | 98.41 |
| Mabs-4(NB)-B | 11587 | 1285 | 97.56 |
| Mabs-5(NB)-B | 1243 | 172 | 97.02 |
| Mabs-6(NB)-B | 344 | 54 | 97.67 |
| Mabs-7(NB)-B | 1484 | 158 | 97.57 |
| Mabs-8(NB)-B | 1033 | 154 | 96.61 |
| Mabs-9(NB)-B | 1421 | 94 | 98.45 |
| **Non-involved site** | | | |
| Mavi-1(FC)-N | 235 | 51 | 86.38 |
| Mavi-2(NB)-N | 848 | 27 | 99.76 |
| Mavi-3(FC)-N | 356 | 79 | 90.45 |
| Mavi-4(NB)-N | 339 | 111 | 86.73 |
| Mavi-5(FC)-N | 1177 | 68 | 99.24 |
| Mavi-7(FC)-N | 2530 | 94 | 98.77 |
| Mavi-8(FC)-N | 394 | 92 | 91.37 |
| Mavi-9(NB)-N | 1173 | 187 | 97.78 |
| Mavi-10(NB)-N | 301 | 114 | 87.38 |
| Mint-2(NB)-N | 469 | 227 | 76.55 |
| Mint-4(FC)-N | 232 | 28 | 98.28 |
| Mint-5(FC)-N | 529 | 168 | 88.66 |
| Mint-6(FC)-N | 360 | 28 | 98.61 |
| Mint-7(FC)-N | 322 | 88 | 91.61 |
| Mint-8(NB)-N | 415 | 87 | 92.05 |
| Mint-10(FC)-N | 223 | 37 | 96.41 |
| Mabs-3(FC)-N | 558 | 127 | 91.04 |
| Mabs-4(NB)-N | 201 | 47 | 96.52 |
| Mabs-5(NB)-N | 520 | 66 | 98.08 |
| Mabs-6(NB)-N | 232 | 45 | 98.28 |
| Mabs-7(NB)-N | 416 | 72 | 92.79 |
| Mabs-8(NB)-N | 207 | 39 | 98.07 |
| Mabs-9(NB)-N | 919 | 130 | 94.34 |

OTU, operational taxonomic unit.
