# Supplementary material for: The lung microbiota in nontuberculous mycobacterial pulmonary disease
Source: PLoS One. 2023 May 26;18(5):e0285143. doi: 10.1371/journal.pone.0285143 (PMC10218745; doi:10.1371/journal.pone.0285143)
Supplement: S2 Table — (DOCX) [file pone.0285143.s005.docx]

**S2 Table.** Antibiotics administered before lung resection of all patients (n = 40).

| **Antibiotics** | **Study population**  **(n = 23)** | **Excluded population (n = 17)** | ***p*-value** |
| --- | --- | --- | --- |
| **Oral agents** |  |  |  |
| Macrolide | 23 (100) | 17 (100) | > 0.999 |
| Duration, months | 22.7 (13.2–38.2) | 15.9 (12.3–28.9) | 0.286 |
| Ethambutol | 15 (65) | 8 (47) | 0.251 |
| Duration, months | 24.8 (15.0–43.1) | 13.7 (3.4–29.4) | 0.164 |
| Rifamycin | 15 (65) | 8 (47) | 0.251 |
| Duration, months | 19.8 (9.9–38.2) | 18.2 (14.3–29.4) | > 0.999 |
| Moxifloxacin | 2 (8) | 0 (0) | 0.499 |
| Duration, months | 1.2, 12.6 | 0.0 | - |
| Clofazimine | 6 (26) | 8 (47) | 0.169 |
| Duration, months | 10.9 (7.1–30.6) | 9.7 (5.3–20.3) | 0.729 |
| Linezolid | 1 (4) | 2 (12) | 0.565 |
| Duration, months | 1.2 | 0.16, 5.97 | > 0.999 |
| **Injectable agents** |  |  |  |
| Aminoglycoside | 15 (65) | 13 (77) | 0.443 |
| Duration, months | 4.6 (1.3–12.6) | 1.2 (0.6–8.9) | 0.145 |
| Cefoxitin | 5 (22) | 7 (41) | 0.185 |
| Duration, months | 1.3 (0.9–7.8) | 0.9 (0.3–1.2) | 0.063 |
| Imipenem | 5 (22) | 6 (35) | 0.477 |
| Duration, months | 0.4 (0.2–7.0) | 0.4 (0.4–0.9) | 0.407 |
| Tigecycline | 0 (0) | 2 (12) | 0.174 |
| Duration, months | 0.0 | 0.2, 0.4 | - |

Data are presented as n (%) or the median (interquartile range).
