# Supplementary material for: The lung microbiota in nontuberculous mycobacterial pulmonary disease
Source: PLoS One. 2023 May 26;18(5):e0285143. doi: 10.1371/journal.pone.0285143 (PMC10218745; doi:10.1371/journal.pone.0285143)
Supplement: S3 Table — (DOCX) [file pone.0285143.s006.docx]

**S3 Table.** Detailed information of antibiotics administered before lung resection of study patients (n = 23).

| **Sample Name** | **Macrolide** | **Duration** | **EMB** | **Duration** | **RFP** | **Duration** | **Mfx** | **Duration** | **CFZ** | **Duration** | **LZD** | **Duration** | **IV**  **AG** | **Duration** | **IV CFX** | **Duration** | **IV IMP** | **Duration** |
| --- | --- | --- | --- | --- | --- | --- | --- | --- | --- | --- | --- | --- | --- | --- | --- | --- | --- | --- |
| Mavi-1(FC) | Yes | 22.7 | Yes | 22.7 | Yes | 22.7 | No | - | No |  | No | - | Yes | 6.0 | No | - | No | - |
| Mavi-2(NB) | Yes | 46.7 | Yes | 46.7 | Yes | 9.9 | No | - | Yes | 11.9 | No | - | No | - | No | - | No | - |
| Mavi-3(FC) | Yes | 55.1 | Yes | 55.1 | Yes | 55.1 | No | - | No | - | No | - | Yes | 15.7 | No | - | No | - |
| Mavi-4(NB) | Yes | 46.8 | Yes | 46.8 | Yes | 46.8 | No | - | No | - | No | - | No | - | No | - | No | - |
| Mavi-5(FC) | Yes | 19.8 | Yes | 19.8 | Yes | 19.8 | No | - | No | - | No | - | Yes | 6.5 | No | - | No | - |
| Mavi-7(FC) | Yes | 24.8 | Yes | 24.8 | Yes | 7.8 | No | - | No | - | No | - | Yes | 3.9 | No | - | No | - |
| Mavi-8(FC) | Yes | 16.2 | Yes | 16.2 | Yes | 16.2 | No | - | No | - | No | - | No | - | No | - | No | - |
| Mavi-9(NB) | Yes | 43.1 | Yes | 43.1 | Yes | 43.1 | No | - | No | - | No | - | No | - | No | - | No | - |
| Mavi-10(NB) | Yes | 38.2 | Yes | 38.2 | Yes | 38.2 | No | - | No | - | No | - | No | - | No | - | No | - |
| Mint-2(NB) | Yes | 34.2 | Yes | 34.2 | Yes | 34.2 | No | - | No | - | No | - | No | - | No | - | No | - |
| Mint-4(FC) | Yes | 4.6 | Yes | 4.6 | Yes | 4.6 | No | - | No | - | No | - | Yes | 4.6 | No | - | No | - |
| Mint-5(FC) | Yes | 15.0 | Yes | 15.0 | Yes | 15.0 | No | - | No | - | No | - | Yes | 2.8 | No | - | No | - |
| Mint-6(FC) | Yes | 12.6 | Yes | 12.6 | Yes | 12.6 | No | - | No | - | No | - | Yes | 12.6 | No | - | No | - |
| Mint-7(FC) | Yes | 7.7 | Yes | 7.7 | Yes | 7.7 | No | - | No | - | No | - | Yes | 8.6 | No | - | No | - |
| Mint-8(NB) | Yes | 13.2 | No | - | No | - | Yes | 12.6 | Yes | 0.7 | No | - | No | - | No | - | No | - |
| Mint-10(FC) | Yes | 31.2 | Yes | 31.2 | Yes | 31.2 | No | - | No | - | No | - | No | - | No | - | No | - |
| Mabs-3(FC) | Yes | 50.4 | No | - | No | - | No | - | Yes | 39.0 | No | - | Yes | 1.3 | Yes | 0.9 | Yes | 0.4 |
| Mabs-4(NB) | Yes | 31.4 | No | - | No | - | No | - | Yes | 27.8 | Yes | 1.2 | Yes | 13.4 | No | - | Yes | 13.4 |
| Mabs-5(NB) | Yes | 14.2 | No | - | No | - | No | - | No | - | No | - | Yes | 14.2 | Yes | 14.1 | Yes | 0.1 |
| Mabs-6(NB) | Yes | 33.8 | No | - | No | - | No | - | Yes | 9.2 | No | - | Yes | 1.3 | Yes | 0.9 | Yes | 0.4 |
| Mabs-7(NB) | Yes | 14.2 | No | - | No | - | No | - | No | - | No | - | Yes | 1.5 | Yes | 1.5 | No | - |
| Mabs-8(NB) | Yes | 1.0 | No | - | No | - | No | - | No | - | No | - | Yes | 0.2 | No | - | Yes | 0.2 |
| Mabs-9(NB) | Yes | 11.0 | No | - | No | - | Yes | 1.2 | Yes | 9.9 | No | - | Yes | 1.3 | Yes | 1.3 | No | - |

Data of duration are presented as months. EMB, ethambutol; RFP, rifamycin; Mfx, moxifloxacin; CFZ, clofazimine; LZD, linezolid; IV, intravenous; AG, aminoglycoside; CFX, cefoxitin; IMP. imipenem
