# Supplementary material for: The lung microbiota in nontuberculous mycobacterial pulmonary disease
Source: PLoS One. 2023 May 26;18(5):e0285143. doi: 10.1371/journal.pone.0285143 (PMC10218745; doi:10.1371/journal.pone.0285143)
Supplement: S4 Table — (DOCX) [file pone.0285143.s007.docx]

**S4 Table.** Proportions of other major respiratory pathogens compared between involved and non-involved sites in study patients (n=23).

| MTP name | ***Stenotrophomonas*** | | ***Haemophilus*** | | ***Burkholderia*** | | ***Pseudomonas*** | |
| --- | --- | --- | --- | --- | --- | --- | --- | --- |
|  | Involved | Non-involved | Involved | Non-involved | Involved | Non-involved | Involved | Non-involved |
|  | Read counts (%) | Read counts (%) | Read counts (%) | Read counts (%) | Read counts (%) | Read counts (%) | Read counts (%) | Read counts (%) |
| Mavi-1(FC) | – | – | 12 (0.04) | 1 (0.43) | 5 (0.02) | 5 (2.13) | 35 (0.12) | – |
| Mavi-2(NB) | 6 (0.45) | – | 2 (0.15) | – | – | – | 32 (2.42) | 90 (10.61) |
| Mavi-3(FC) | 12 (0.27) | 1 (0.28) | 5 (0.11) | 1 (0.28) | – | – | 57 (1.28) | 28 (7.87) |
| Mavi-4(NB) | – | – | 6 (0.08) | 2 (0.59) | 6 (0.08) | – | 263 (3.55) | – |
| Mavi-5(FC) | – | 15 (1.27) | 21 (0.24) | – | – | – | 26 (0.30) | 1 (0.09) |
| Mavi-7(FC) | 1 (0.04) | 3 (0.64) | 1 (0.04) | 1 (0.03) | – | – | 6 (0.21) | – |
| Mavi-8(FC) | 1 (0.07) | – | 1 (0.07) | 1 (0.25) | – | – | – | 4 (1.02) |
| Mavi-9(NB) | 6 (2.71) | 12 (1.02) | 6 (2.71) | – | – | – | 27 (12.22) | 4 (0.34) |
| Mavi-10(NB) | 6 (1.97) | 2 (0.66) | – | – | – | – | – | – |
| Mint-2(NB) | 1 (0.01) | – | 96 (1.29) | – | 5 (0.07) | – | 15 (0.20) | 2 (0.43) |
| Mint-4(FC) | 12 (0.11) | 2 (0.86) | 5 (0.05) | – | 5 (0.05) | – | 932 (8.61) | – |
| Mint-5(FC) | 9 (0.21) | – | 8 (0.29) | 4 (0.76) | 11 (0.26) | – | 33 (0.78) | 3 (0.57) |
| Mint-6(FC) | – | – | 12 (0.28) | 4 (1.11) | 1 (0.02) | – | 100 (2.33) | 4 (1.11) |
| Mint-7(FC) | – | – | 20 (0.21) | – | 7 (0.07) | – | 6 (0.06) | 11 (3.42) |
| Mint-8(NB) | 2 (0.03) | – | 27 (0.36) | 8 (1.93) | 2 (0.03) | – | 1 (0.01) | 3 (0.72) |
| Mint-10(FC) | 13 (1.24) | 22 (9.87) | – | – | – | – | – | – |
| Mabs-3(FC) | – | – | 1 (0.02) | 6 (1.07) | – | – | 148 (3.47) | – |
| Mabs-4(NB) | – | 11 (5.47) | 36 (0.31) | – | 6 (0.05) | – | 77 (0.66) | – |
| Mabs-5(NB) | – | 24 (4.62) | 12 (0.97) | – | 4 (0.32) | – | – | – |
| Mabs-6(NB) | 8 (2.33) | 10 (4.31) | – | – | – | – | 2 (0.58) | 5 (2.16) |
| Mabs-7(NB) | – | 1 (0.24) | 4 (0.27) | 1 (0.24) | 1 (0.07) | – | – | 2 (0.48) |
| Mabs-8(NB) | 5 (0.48) | 17 (8.12) | – | 14 (6.76) | 2 (0.19) | – | – | 2 (0.97) |
| Mabs-9(NB) | 14 (0.99) | – | – | – | – | – | – | – |

MTP, microbial taxonomic profile; FC, fibrocavitary; NB, nodular bronchiectatic; –, negative.
