# Supplementary material for: The lung microbiota in nontuberculous mycobacterial pulmonary disease
Source: PLoS One. 2023 May 26;18(5):e0285143. doi: 10.1371/journal.pone.0285143 (PMC10218745; doi:10.1371/journal.pone.0285143)
Supplement: S5 Table — (DOCX) [file pone.0285143.s008.docx]

**S5 Table.** Taxonomic biomarker analysis for genera compared between involved and non-involved sites in study patients (n=23).

| **Taxon name** | **Involved site (%)** | **Non-involved site (%)** | **LDA effect size** | ***p*-value** | ***q-v*alue**^*^ |
| --- | --- | --- | --- | --- | --- |
| **Genus** |  |  |  |  |  |
| *Acinetobacter* | 0.64 | 4.53 | 4.27 | < 0.001 | 0.002 |
| *Limnohabitans* | 3.39 | 0.11 | 4.17 | < 0.001 | 0.027 |
| *Faecalibacterium* | 3.29 | 0.90 | 4.13 | 0.010 | 0.138 |
| *Rahnella* | 2.33 | 0.05 | 4.05 | < 0.001 | 0.029 |
| *Blautia* | 1.71 | 0.62 | 3.78 | 0.024 | 0.231 |
| *Ruminococcus* | 1.07 | 0.12 | 3.72 | 0.003 | 0.079 |
| *Oscillibacter* | 1.56 | 0.53 | 3.70 | 0.004 | 0.093 |
| *Lachnospira* | 0.98 | 0.11 | 3.67 | < 0.001 | 0.036 |
| *Methylotenera* | 0.97 | 0.07 | 3.60 | 0.003 | 0.085 |
| *Enhydrobacter* | 0.65 | 1.14 | 3.55 | 0.015 | 0.189 |
| *Aquabacterium* | 1.02 | 0.20 | 3.54 | 0.019 | 0.189 |
| *Roseburia* | 0.76 | 0.16 | 3.51 | 0.007 | 0.134 |
| *Nanopelagicus* | 0.48 | 0.06 | 3.44 | 0.003 | 0.093 |
| *Alistipes* | 0.88 | 0.63 | 3.36 | 0.025 | 0.234 |
| *Hydrogenophaga* | 0.52 | 0.03 | 3.36 | 0.001 | 0.056 |
| *Fusicatenibacter* | 0.48 | 0.12 | 3.35 | 0.002 | 0.070 |
| *Acidibacter* | 0.42 | 0.09 | 3.25 | 0.003 | 0.078 |
| *Flavobacterium* | 0.49 | 0.13 | 3.25 | 0.001 | 0.049 |
| *Agathobacter* | 0.41 | 0.18 | 3.23 | 0.007 | 0.136 |
| *Pseudolabrys* | 0.43 | 0.14 | 3.21 | 0.022 | 0.214 |
| *Anaerostipes* | 0.48 | 0.22 | 3.19 | 0.014 | 0.189 |
| *Megamonas* | 0.28 | 0.01 | 3.19 | <0.001 | 0.008 |
| *Parabacteroides* | 0.34 | 0.12 | 3.16 | 0.012 | 0.169 |
| *Gaiella* | 0.32 | 0.11 | 3.16 | 0.001 | 0.045 |
| *Subdoligranulum* | 0.26 | 0.03 | 3.10 | <0.001 | 0.020 |
| *Rheinheimera* | 0.31 | 0.03 | 3.09 | <0.001 | 0.017 |
| *Tepidisphaera* | 0.25 | 0.08 | 3.09 | 0.003 | 0.093 |
| *Dorea* | 0.28 | 0.06 | 3.08 | 0.001 | 0.043 |
| *Collinsella* | 0.27 | 0.07 | 3.06 | <0.001 | 0.032 |
| *Enterococcus* | 0.33 | 0.14 | 3.05 | 0.013 | 0.180 |
| *Undibacterium* | 0.23 | 0.01 | 3.05 | 0.001 | 0.052 |
| *Bacillus* | 0.35 | 0.11 | 3.04 | 0.004 | 0.093 |
| *Bradyrhizobium* | 0.60 | 0.39 | 3.03 | 0.008 | 0.138 |
| *Phascolarctobacterium* | 0.25 | 0.08 | 3.01 | 0.001 | 0.039 |

LEfSe, linear discriminant analysis effect size; LDA, linear discriminant analysis. LEfSe analysis included all taxa, including taxa with proportions <1%. **^†^**Adjusted p–value; the Benjamini–Hochberg false discovery rate was applied to correct for multiple testing, and values of less than 0.05 were considered significant.
