# Supplementary material for: The lung microbiota in nontuberculous mycobacterial pulmonary disease
Source: PLoS One. 2023 May 26;18(5):e0285143. doi: 10.1371/journal.pone.0285143 (PMC10218745; doi:10.1371/journal.pone.0285143)
Supplement: S6 Table — (DOCX) [file pone.0285143.s009.docx]

**S6 Table.** Predominant genera in non-involved site compared to involved site identified by LEfSe analysis (n=23).

| MTP name | ***Acinetobacter*** | | ***Enhydrobacter*** | |
| --- | --- | --- | --- | --- |
|  | Involved | Non-involved | Involved | Non-involved |
|  | Read counts (%) | Read counts (%) | Read counts (%) | Read counts (%) |
| Mavi-1(FC) | 2 (0.01) | 22 (9.36) | – | 5 (2.13) |
| Mavi-2(NB) | 3 (0.23) | 19 (2.24) | – | 7 (0.83) |
| Mavi-3(FC) | 13 (0.29) | 42 (11.80) | – | 3 (0.84) |
| Mavi-4(NB) | 15 (0.20) | 36 (10.62) | 3 (0.04) | – |
| Mavi-5(FC) | 4 (0.05) | 40 (3.40) | – | 11 (0.94) |
| Mavi-7(FC) | – | 20 (0.79) | – | 1 (0.04) |
| Mavi-8(FC) | – | 36 (9.14) | – | 14 (3.55) |
| Mavi-9(NB) | 4 (1.81) | 64 (5.46) | 33 (14.93) | 3 (0.26) |
| Mavi-10(NB) | 3 (0.98) | 38 (12.62) | 3 (0.98) | – |
| Mint-2(NB) | 19 (0.25) | 24 (5.12) | – | 3 (0.64) |
| Mint-4(FC) | 4 (0.04) | 17 (7.33) | – | – |
| Mint-5(FC) | 9 (0.21) | 1 (0.19) | – | – |
| Mint-6(FC) | 4 (0.09) | 26 (7.22) | – | – |
| Mint-7(FC) | 5 (0.05) | 35 (10.87) | 1 (0.01) | 5 (1.55) |
| Mint-8(NB) | – | 41 (9.88) | – | 7 (1.69) |
| Mint-10(FC) | 11 (1.05) | 62 (27.8) | – | – |
| Mabs-3(FC) | – | 53 (9.50) | 6 (0.14) | 19 (3.41) |
| Mabs-4(NB) | 4 (0.03) | 48 (23.88) | 6 (0.05) | 1 (0.50) |
| Mabs-5(NB) | 4 (0.32) | 18 (3.46) | – | 21 (4.04) |
| Mabs-6(NB) | 65 (18.90) | 9 (3.88) | – | 18 (7.76) |
| Mabs-7(NB) | 7 (0.47) | 54 (12.98) | 3 (0.20) | – |
| Mabs-8(NB) | – | 8 (3.86) | 4 (0.39) | – |
| Mabs-9(NB) | 37 (2.60) | 42 (4.57) | 3 (0.21) | 8 (0.87) |

MTP, microbial taxonomic profile; FC, fibrocavitary; NB, nodular bronchiectatic; –, negative.
