# Supplementary material for: The lung microbiota in nontuberculous mycobacterial pulmonary disease
Source: PLoS One. 2023 May 26;18(5):e0285143. doi: 10.1371/journal.pone.0285143 (PMC10218745; doi:10.1371/journal.pone.0285143)
Supplement: S7 Table — (DOCX) [file pone.0285143.s010.docx]

**S7 Table.** Taxonomic biomarker analysis for genera compared between NB (n=12) and FC (n=11) forms in involved site.

| **Taxon name** | **NB form (%)** | **FC form (%)** | **LDA effect size** | ***p*-value** | ***q-v*alue^*^** |
| --- | --- | --- | --- | --- | --- |
| **Genus** |  |  |  |  |  |
| *Mycobacterium* | 0.42 | 11.24 | 4.58 | 0.018 | 0.492 |
| *Bacteroides* | 1.72 | 8.69 | 4.58 | 0.009 | 0.492 |
| *Faecalibacterium* | 1.10 | 5.69 | 4.39 | 0.008 | 0.492 |
| *Aquabacterium* | 1.94 | 0.03 | 3.99 | 0.023 | 0.492 |
| *Blautia* | 0.90 | 2.59 | 3.96 | 0.016 | 0.492 |
| *Ruminococcus* | 0.30 | 1.90 | 4.58 | 0.018 | 0.492 |
| *Oscillibacter* | 0.89 | 2.29 | 4.58 | 0.009 | 0.492 |
| *Lachnospira* | 0.33 | 1.69 | 4.39 | 0.008 | 0.492 |
| *Acinetobacter* | 1.17 | 0.06 | 3.99 | 0.023 | 0.492 |
| *Roseburia* | 0.24 | 1.34 | 3.96 | 0.016 | 0.492 |
| *Bifidobacterium* | 0.86 | 1.91 | 3.92 | 0.022 | 0.492 |
| *Pseudoflavonifractor* | 0.20 | 0.97 | 3.89 | 0.041 | 0.492 |
| *Fusicatenibacter* | 0.22 | 0.76 | 3.87 | 0.008 | 0.492 |
| *Parabacteroides* | 0.12 | 0.58 | 3.77 | 0.048 | 0.492 |
| *Undibacterium* | 0.43 | 0.02 | 3.76 | 0.013 | 0.492 |
| *Agathobacter* | 0.23 | 0.62 | 3.76 | 0.021 | 0.492 |
| *Caproiciproducens* | 0.05 | 0.40 | 3.61 | 0.002 | 0.492 |
| *Dorea* | 0.15 | 0.42 | 3.49 | 0.011 | 0.492 |
| *Parasutterella* | 0.05 | 0.32 | 3.41 | 0.022 | 0.492 |
| *Massilia* | 0.37 | 0.01 | 3.36 | 0.034 | 0.492 |
| *Nitrospira* | 0.28 | 0.02 | 3.34 | 0.018 | 0.492 |
| *Subdoligranulum* | 0.13 | 0.39 | 3.26 | 0.019 | 0.492 |
| *Phascolarctobacterium* | 0.13 | 0.38 | 3.20 | 0.021 | 0.492 |
| *Agathobaculum* | 0.10 | 0.32 | 3.18 | 0.012 | 0.492 |
| *Streptococcus* | 0.42 | 0.51 | 3.18 | 0.000 | 0.492 |

LEfSe, linear discriminant analysis effect size; LDA, linear discriminant analysis; NB, nodular bronchiectatic; FC, fibrocavitary. LEfSe analysis included all taxa, including taxa with proportions <1%. **^†^**Adjusted p–value; the Benjamini–Hochberg false discovery rate was applied to correct for multiple testing, and values of less than 0.05 were considered significant.
